# Dysplasia-Carcinoma Transition Specific Transcripts in Colonic Biopsy Samples

**DOI:** 10.1371/journal.pone.0048547

**Published:** 2012-11-14

**Authors:** Orsolya Galamb, Barnabás Wichmann, Ferenc Sipos, Sándor Spisák, Tibor Krenács, Kinga Tóth, Katalin Leiszter, Alexandra Kalmár, Zsolt Tulassay, Béla Molnár

**Affiliations:** 1 2nd Department of Medicine, Semmelweis University, Budapest, Hungary; 2 Molecular Medicine Research Unit, Hungarian Academy of Sciences, Budapest, Hungary; 3 1st Department of Pathology and Experimental Cancer Research, Semmelweis University, Budapest, Hungary; University of Munich, Germany

## Abstract

**Background:**

The early molecular detection of the dysplasia-carcinoma transition may enhance the strength of diagnosis in the case of colonic biopsies. Our aims were to identify characteristic transcript sets in order to develop diagnostic mRNA expression patterns for objective classification of benign and malignant colorectal diseases and to test the classificatory power of these markers on an independent sample set.

**Methodology/Principal Findings:**

Colorectal cancer (CRC) and adenoma specific transcript sets were identified using HGU133plus2 microarrays and 53 biopsies (22 CRC, 20 adenoma and 11 normal). Ninety-four independent biopsies (27 CRC, 29 adenoma and 38 normal) were analyzed on microarrays for testing the classificatory power of the discriminatory genes. Array real-time PCR validation was done on 68 independent samples (24 CRC, 24 adenoma and 20 normal). A set of 11 transcripts (including CXCL1, CHI3L1 and GREM1) was determined which could correctly discriminate between high-grade dysplastic adenoma and CRC samples by 100% sensitivity and 88.9% specificity. The discriminatory power of the marker set was proved to be high on independent samples in both microarray and RT-PCR analyses. 95.6% of original and 94.1% of cross-validated samples was correctly classified in discriminant analysis.

**Conclusions/Significance:**

The identified transcripts could correctly characterize the dysplasia-carcinoma transition in biopsy samples, also on a large independent sample set. These markers can establish the basis of gene expression based diagnostic classification of colorectal cancer. Diagnostic RT-PCR cards can become part of the automated routine procedure.

## Introduction

Colorectal cancer (CRC) is the third most common cancer type and the second leading cause of cancer related mortality in the Western countries [1]. It is thought to develop slowly via a progressive accumulation of genetic mutations, epigenetic and gene expression alterations; recurrence risk and overall mortality of CRC is closely related to the stage of disease at time of primary diagnosis [2]. Histological differentiation of high-grade dysplasia from well-differentiated carcinoma is often difficult, even in the case of correct sampling. A molecular test for CRC should be able to identify the disease at early stage with high specificity and sensitivity, thus enabling effective treatment from the onset before the disease progresses.

Microarray analyses have already been applied to investigate gene expression changes in many cancer types including CRC [3]–[14]. Gene expression marker sets can be identified by whole genomic expression profiling of colonic biopsy samples which would establish the basis of the molecular biological classification of colorectal diseases. Recent microarray studies determined mRNA expression patterns related to:

– colorectal carcinogenesis, progression and metastatic development [3]–[8].
– different subtypes of CRC with diverse clinicopathological parameters [4], [8]–[10].
– limited number of experiments focusing on molecular-based prognosis [11].

The whole genomic microarrays are suitable for high-throughput marker selection, but the high costs and time-consuming execution make their prospective introduction as a diagnostic tool difficult. Furthermore, the evaluation of the huge amount of data collected by microarray analyses requires an extensive bioinformatics with multivariate statistical methods.

However, the newer generation of real-time PCR instruments available with multiplex arrays enables the testing and diagnostic utilization of mRNA expression microarray data. These quantitative array real-time PCRs with 384-well plates give an opportunity for testing the selected marker panels on a large set of independent samples allowing the measuring of the expression of more than hundred genes simultaneously. For the sake of flexibility quantitative RT-PCR with multiple transcript panels are custom-designed [15]. Universal ProbeLibrary probes from Roche use a unique nucleotide chemistry called LNA (Locked Nucleic Acid), which allows very short (8–9 bases) oligonucleotides to be efficient hybridization probes in real-time PCR assays. Optimized primer pairs and UPL probes can make the array RT-PCR a robust, reliable, quick and cost effective gene expression analyzing method which can be suitable for daily diagnostic utilization in the future.

Traditional histology may suffer from sampling bias due to biopsy orientation problems, therefore, critical areas including aberrant crypt foci, dysplastic areas or in situ carcinoma may remain hidden. Molecular based discrimination using mRNA expression can represent the whole sample to avoid this bias and support pathologists in coping with their growing workload of early cancer screening. Furthermore, mRNA expression can reveal functional information beyond microscopy related to the biological behavior, tumor invasion, metastasic spread and therapeutic target expression in colorectal cancer.

In this study, we applied whole genomic microarray analysis in order to identify gene expression profile alterations focusing on the dysplastic adenoma-carcinoma transition. Our aims were to identify characteristic transcript sets in order to develop diagnostic mRNA expression patterns for objective classification of benign and malignant colorectal diseases and to test the classificatory power of these markers on an independent sample set.

## Materials and Methods

### Patients and samples

After informed consent of untreated patients, colon biopsy samples were taken during endoscopic intervention and stored in RNALater Reagent (Qiagen Inc, Germantown, US) at –80°C. Altogether 147 biopsy specimen (53/original set/and additionally 94 fresh frozen/independent set/samples) were analyzed in our study. Total RNA was extracted and Affymetrix microarray analysis was performed on biopsies of patients with tubulovillous/villous adenomas (n = 29, 13 high-grade dysplastic and 16 with low-grade dysplasia), colorectal adenocarcinoma (n = 27, 14 early and 13 advanced CRC) and of healthy normal controls (n = 38). Fifty three microarrays (11 normal, 20 adenoma, 22 CRC) had been hybridized earlier (original samples set), their data files were used in a previous studies using different comparisons [12]–[14] and are available in the Gene Expession Omnibus database (series accession numbers: GSE4183 and GSE10714), while GSE37364 accession number refers to the data files of newly hybridized 94 microarrays (independent sample set). The diagnostic groups and the number of patients in each group are represented in Table 1. Detailed patient specification is described in Table S1. The study involves human subjects. Therefore the study was approved by the Regional and Institutional Committee of Science and Research Ethics (TUKEB Nr.: 69/2008. Semmelweis University Regional and Institutional Committee of Science and Research Ethics, Budapest, Hungary). Written informed consent was obtained from all patients.

**Table 1:** Number of patients per disease group participating in the study.

Group	Original set	Independent set	
	Affymetrix microarrays (GSE4183, GSE10714)	Affymetrix microarrays GSE37364	Array real-time PCR
Adenoma with low-grade dysplasia	9	16	13
High-grade dysplastic adenoma	11	13	11
CRC Dukes A–B	10	14	10
CRC Dukes C–D	12	13	10
CRC with unknown stage	-	-	4
Healthy Control	11	38	20
Total patient numbers	53	94	68

### mRNA expression microarray analysis

Total RNA was extracted using the RNeasy Mini Kit (Qiagen) according to the manufacturer's instructions. Quantity and quality of the isolated RNA were tested by measuring the absorbance and capillary gelelectrophoresis using the 2100 Bioanalyzer and RNA 6000 Pico Kit (Agilent Inc, Santa Clara, US). Biotinylated cRNA probes were synthesized from 4,82±0,60 µg total RNA and fragmented using the One-Cycle Target Labeling and Control Kit (http://www.affymetrix.com/support/downloads/manuals/ expression_analysis_technical_manual.pdf) according to the Affymetrix description. Ten µg of each fragmented cRNA sample were hybridized into HGU133 Plus2.0 array (Affymetrix) at 45°C for 16 hours. The slides were washed and stained using Fluidics Station 450 and an antibody amplification staining method according to the manufacturer's instructions. The fluorescent signals were detected by a GeneChip Scanner 3000.

### Statistical evaluation of mRNA expression profiles

Quality control analyses were performed according to the suggestions of the Tumour Analysis Best Practices Working Group [16]. Scanned images were inspected for artifacts, percentage of present calls (>25%) and control of the RNA degradation were evaluated. Based on the evaluation criteria all biopsy measurements fulfilled the minimal quality requirements. The Affymetrix expression arrays were pre-processed by gcRMA with quantile normalization and median polish summarization. The datasets are available in the Gene Expression Omnibus databank for further analysis (http://www.ncbi.nlm.nih.gov/geo/), series accession numbers: GSE4183, GSE10714).

Differentially expressed genes were identified by Significance Analysis of microarrays (SAM) method between different diagnostic groups. The nearest shrunken centroid method (Prediction Analysis for miroarrays – PAM) was applied for sample classification from gene expression data. The pre-processing, data mining and statistical steps were performed using R-environment with Bioconductor libraries. Hierarchical cluster analysis represents on each comparisons of correlation. Logistic regression was applied to analyze dependence of binary diagnostic variables (represented 0 as control, 1 as disease). Discriminant and principal component analysis were also performed. In the discriminant analysis, leave-one out classification was applied for cross-validation.

### Array real-time PCR

Commercially available real-time PCR assays were applied for expression measuring of 11 discriminatory transcripts (www.roche-applied-science.com). The list of the real-time ready assays can be seen in the Table 2. Gene specific forward and reverse primers and fluorescently labeled hydrolysis probes from Universal ProbeLibrary (F. Hoffmann-La Roche Ltd., Switzerland, Basel) were lyophilized into wells of 384-well PCR plates. Using Transcriptor First Strand cDNA Synthesis Kit (Roche), 2.5 µg total RNA from 20 healthy, 24 adenoma, 24 CRC biopsy samples were reverse transcribed (Table 1). The quality of the cDNA samples was checked by real-time PCR for SDHA (succinate dehydrogenase complex, subunit A, flavoprotein) housekeeping gene. The expression analysis of the selected genes was performed from 5 ng/sample cDNA template, using the newly designed array real-time PCR cards and LightCycler 480 Probes Master (Roche). The measurements were performed using a LightCycler 480 instrument as described in the products User Guide (http://www.roche-applied-science.com). After enzyme activation and denaturation at 95°C for 10 min, 45 PCR cycles were performed (denaturation at 95°C for 10 sec, annealing and extension at 60°C for 30 sec and signal detection at 72°C for 1 sec). In order to select the most appropriate reference gene, seven different housekeeping genes (glyceraldehyde-3-phosphate dehydrogenase (GAPDH), β2-microglobulin (B2M), beta-actin (ACTB), hypoxanthine phosphoribosyltransferase 1 (HPRT1), ribosomal protein L13a (RPL13A), 18S ribosomal RNA (18S), tyrosine 3-monooxygenase/tryptophan 5-monooxygenase activation protein, zeta polypeptide (YWHAZ)) were used on the real-time PCR array.

**Table 2:** Real-time ready assays applied in RT-PCR validation.

Assay ID	Gene Symbol	Gene name	Amplicon length	Position	Intron spanning
103015	CA7	carbonic anhydrase VII	77	416–492	+
100950	IL1B	interleukin 1, beta	87	162–248	+
103133	IL1RN	interleukin 1 receptor antagonist	76	343–418	+
103136	IL8	interleukin 8	92	879–970	−
103109	GREM1	gremlin 1	111	144–254	+
105522	CXCL1	chemokine (C-X-C motif) ligand 1	105	340–444	−
103070	CXCL2	chemokine (C-X-C motif) ligand 2	95	431–525	+
103045	COL12A1	collagen, type XII, alpha 1	66	2287–2352	+
103035	CHI3L1	chitinase 3-like 1	76	433–507	+
103210	SLC7A5	solute carrier family 7, member 5	72	1500–1571	+
103167	MMP3	matrix metallopeptidase 3	110	1210–1319	+
101128	GAPDH	glyceraldehyde-3-phosphate dehydrogenase	112	30–141	+
102065	B2M	beta-2-microglobulin	76	360–435	+
102488	ACTB	actin, beta	102	1047–1148	+
102079	HPRT1	hypoxanthine phosphoribosyltransferase 1	102	218–319	+
102119	RPL13A	ribosomal protein L13a	124	317–440	+
104092	RN18S1	RNA, 18S ribosomal 1, 18S ribosomal RNA	73	982–154	−
102125	YWHAZ	Top of Form tyrosine 3-monooxygenase/tryptophan 5-monooxygenase activation protein, zeta polypeptide	130	453–582	+

### Statistical evaluation of RT-PCR results

Relative quantifications of the gene expression were performed and the fold change values were calculated using the ΔΔCT method. The threshold cycle (CT) of the 18S ribosomal RNA endogenous control was used to normalize target gene expression (ΔCT) to correct for experimental variation. Logistic regressions were applied to analyze dependence of binary diagnostic variables (represented 0 as control, 1 as disease) on the ΔCt values from the training set. When P (probability of a patient sample) is diagnosed as “diseased,” then a function X =  logit (P) can be defined as follows:
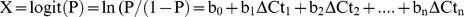



Maximum-likelihood fitting method was used to obtain the (empirical) coefficients {bi} that define the relationship between X and the experimental measurements {ΔCti}. The {bi} values were obtained using MedCalc software program (MedCalc Software). Receiving operating characteristic (ROC) curve analysis was applied to evaluate the discriminatory power of the gene panels [17].

Discriminant and principal component analysis were performed. Discriminant analysis was used primarily in order to predict membership of distinct groups. As a result “Classification results” tables were prepared showing a summary for subjects according to number and percent classified correctly and incorrectly. Leave-one-out classification as cross-validation method was applied. Effective utilization of the discriminant function analysis allowed for a higher percentage of correct estimates from the set of data in the classification table to be possible [18].

Further to this, Principal Components Analysis (PCA) was used as a data dimensionality reduction method which performed a covariance analysis between the determined factors and allowed viewing of multiple datasets into two or three-dimensional figure [19].

### Independent Gene Expression Omnibus datasets

Microarray datasets with HGU133 Plus2.0 experiments obtained from colonic biopsy/tissue samples collected by other research groups were downloaded from Gene Expression Omnibus (GEO) database (dataset IDs: GSE8671 [20], GSE18105 [21]). Our discriminatory marker panel from the study was then tested on the downloaded datasets, and discriminatory efficacy was determined using principal component analysis (PCA) and hierarchical cluster analysis.

## Results

### Discriminatory marker set identified by microrray analysis on the original sample set

Using the original sample group (53 microarrays from 11 normal, 22 CRC and 20 adenoma samples), a set of 11 differentiating transcripts was identified. This set could correctly discriminate not only between the diseased and the normal samples, but could also discriminate between adenoma and CRC samples. Table 3 represents the best discriminating transcripts with fold change values.

**Table 3:** The set of 11 discriminatory transcripts.

			Microarray – original sample set (53)			Microarray – independent sample set (94)			RT-PCR independent sample set (68)		
Affymetrix ID	Gene Symbol	Gene name	Log_2_FC (AD vs. N)	Log_2_FC (CRC vs. N)	Log_2_FC (CRC vs. AD)	Log_2_FC (AD vs. N)	Log_2_FC (CRC vs. N)	Log_2_FC (CRC vs. AD)	Log_2_FC (AD vs. N)	Log_2_FC (CRC vs. N)	Log_2_FC (CRC vs. AD)
207504_at	CA7	carbonic anhydrase VII	−6.3	−4.9	1.5	−5.4	−5.1	0.2	−5.8	−4.1	1.7
39402_at	IL1B	interleukin 1, beta	3.4	4.5	1.1	2.2	6.1	3.9	1.7	4.7	3.0
212657_s_at	IL1RN	interleukin 1 receptor antagonist	3.3	4.7	1.4	1.7	5.1	3.4	1.0	3.3	2.3
202859_x_at	IL8	interleukin 8	5.2	6.6	1.4	4.1	9.0	4.8	2.2	4.4	2.2
218469_at	GREM1	gremlin 1	0.2	4.2	4.0	−0.9	3.0	3.9	2.4	4.5	2.1
204470_at	CXCL1	chemokine (C-X-C motif) ligand 1	5.0	5.1	0.1	4.1	6.3	2.2	−0.04	2.7	2.7
209774_x_at	CXCL2	chemokine (C-X-C motif) ligand 2	4.6	4.1	−0.5	3.7	5.7	2.0	1.1	4.6	3.5
225664_at	COL12A1	collagen, type XII, alpha 1	2.5	3.8	1.4	1.4	3.9	2.5	1.0	3.4	2.4
209395_at	CHI3L1	chitinase 3-like 1	3.4	5.3	1.9	3.3	6.3	3.0	1.4	6.0	4.6
201195_s_at	SLC7A5	solute carrier family 7, member 5	4.6	4.2	−0.4	3.2	4.7	1.5	1.8	6.3	4.5
205828_at	MMP3	matrix metallopeptidase 3	8.2	9.7	1.5	4.0	8.4	4.4	1.8	3.2	1.4

FC  =  fold change.

**Figure 1:**
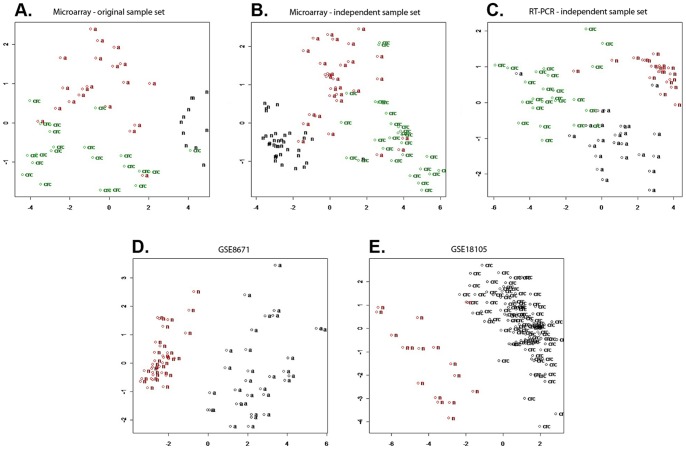
Discriminatory power of the classifier set with 11 transcripts – Principal component analysis. A. Original sample set (53 samples, microarray) B. Independent sample set (94 samples, microarray) C. Independent sample set (68 samples, RT-PCR) D. GSE8671 (64 samples, microarray) E. GSE18105 (111 samples, microarray) a =  adenoma, n =  normal, crc  =  colorectal cancer.

Using PCA the marker set shows clear separation of adenoma, normal and CRC cases (Figure 1A). Using discriminant analysis, 96.2% of originally grouped cases were correctly classified, while 83.0% of cross-validated grouped cases were correctly classified (Table 4).

**Table 4:** Discriminant analysis results of the 11 classificatory transcripts.

			Original sample set (n = 53microarrays)				Independent sample set(n = 94 microarrays)				Independent sample set (n = 68 RT-PCR reactions)			
			*Normal*	*Adenoma*	*CRC*	*Total*	*Normal*	*Adenoma*	*CRC*	*Total*	*Normal*	*Adenoma*	*CRC*	*Total*
*Original*	*Count*	*Normal*	11	0	0	11	38	0	0	38	20	0	0	20
		*Adenoma*	0	20	0	20	2	25	2	29	1	22	1	24
		*CRC*	1	1	20	22	0	2	25	27	1	0	23	24
	*Percentage*													
		*Normal*	100	0	0	100	100	0	0	100	100	0	0	100
		*Adenoma*	0	100	0	100	6.9	86.2	6.9	100	4.2	91.7	4.2	100
		*CRC*	4.5	4.5	90.9	100	0	7.4	92.6	100	4.2	0	95.8	100
*Cross-validated*	*Count*	*Normal*	11	0	0	11	37	0	1	38	20	0	0	20
		*Adenoma*	2	15	3	20	2	25	2	29	1	21	2	24
		*CRC*	1	3	18	22	1	2	24	27	1	0	23	24
	*Percentage*													
		*Normal*	100	0	0	100	97.4	0	2.6	100	100	0	0	100
		*Adenoma*	10	75	15	100	6.9	86.2	6.9	100	4.2	87.5	8.3	100
		*CRC*	4.5	13.6	81.8	100	3.9	7.4	88.9	100	4.2	0	95.8	100

**Figure 2:**
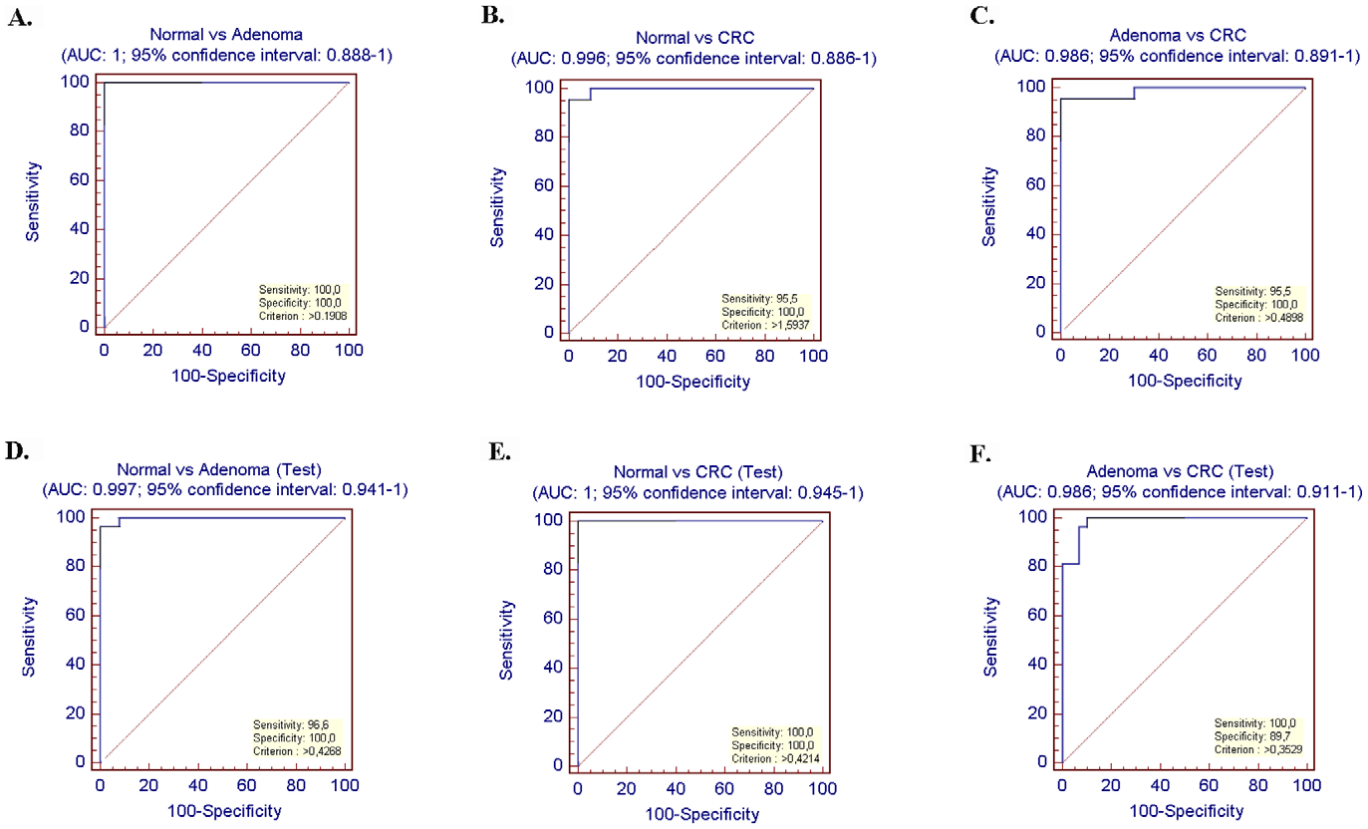
ROC statistic results of original sample group of microarray (53 samples) (A–C), independent sample group of microarray (94 samples) (D–F). The applied multiple logistic regression equations were applied on the different datasets.

When paired comparisons were performed using the 11 differentiating markers, ROC analysis was applied. Normal and adenoma samples could be discriminated by 100% specificity and 100% sensitivity. The specificity was 100% and the sensitivity was 95.5% when CRC and normal biopsy samples were separated. Adenoma and CRC samples could be also classified by considerably high specificity and sensitivity (specificity: 100%, sensitivity: 95.5) (Figure 2 A–C). Youden indices were calculated in order to determinate discriminatory strength. These values vary between 0.91 and 1.

**Figure 3:**
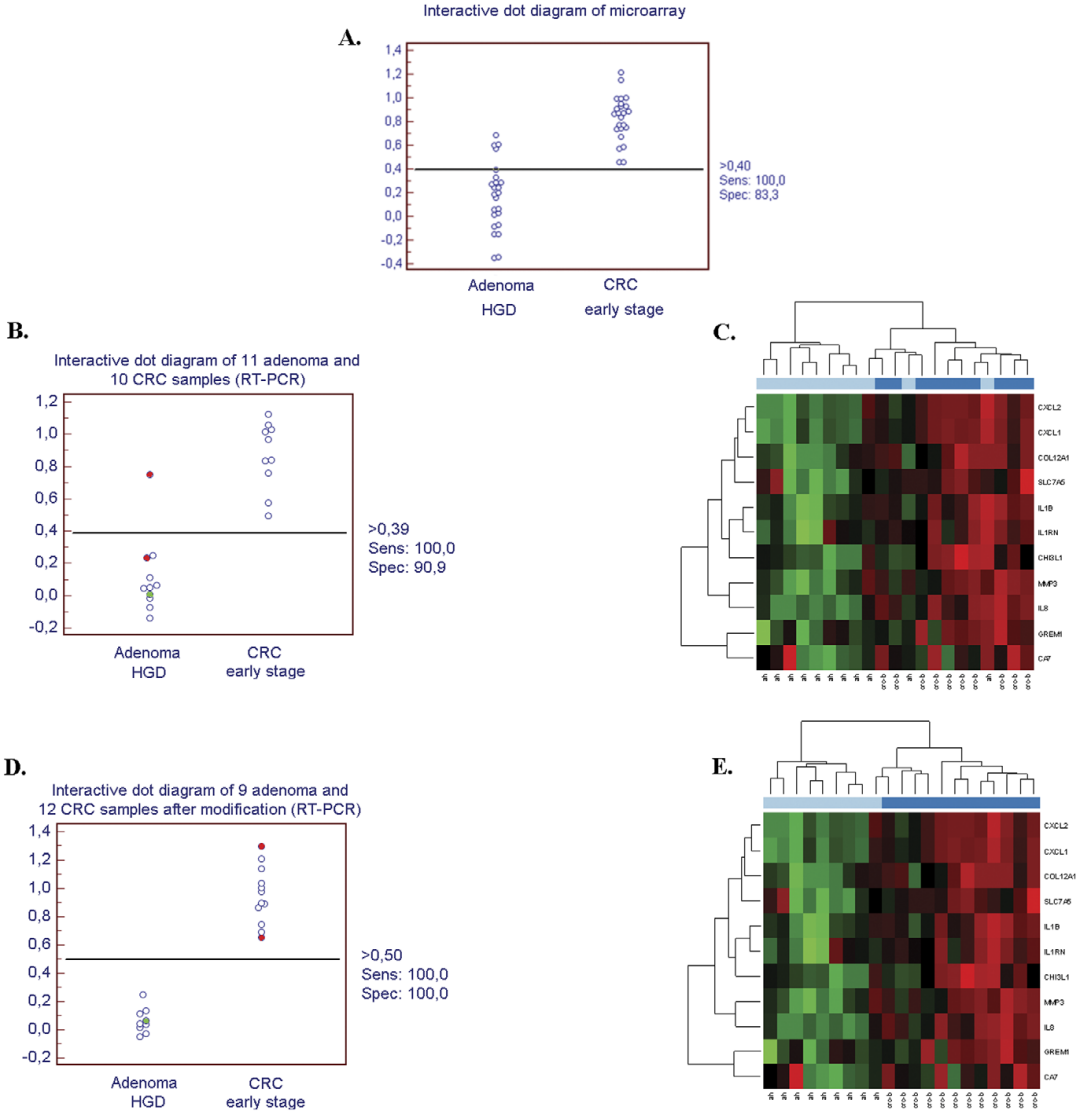
Separation of high-grade dysplastic adenoma and early cancer samples using the set of 11 transcripts. A. Microarray B. Real-time PCR C. Heat map of real-time PCR D. Real-time PCR considering the changes in the diagnosis E. Heat map of real time PCR considering the changes in the diagnosis; Adenoma HGD =  high-grade dysplastic adenoma, CRC early stage =  colorectal cancer early stage.

Using the set of the 11 markers resulted in clear differentiation between high-grade dysplastic adenoma (n = 11) and early stage CRC (n = 10) biopsy samples (specificity: 90.9%, sensitivity: 100%) (Figure 3B).

### Testing of the identified marker set with 11 classificatory genes on independent samples

#### Additional microarrays

Principal component analysis of microarray data from independent biopsy samples resulted in distinct clusters of normal, adenoma and CRC cases with small overlaps between the diagnostic groups (Figure 1B). In discriminant analysis 93.6% of the original samples and 91.5% of cross-validated samples were correctly classified (Table 4).

In paired comparison, according to the discriminatory set with 11 classifiers, the independent CRC and normal samples could be clearly separated. The sensitivity was 100%, the specificity was 100%. Using the discriminatory panel, independent adenoma and healthy samples could be distinguished with 100% specificity and 96.6% sensitivity. The marker set was suitable for classification of the independent benign and malignant colon samples with 89.7% specificity and 100% sensitivity (Figure 2 D–F).

The independent high-grade dysplastic adenoma (n = 13) and early stage CRC (n = 14) biopsy samples could be discriminated by 92.3% specificity and 100% sensitivity. Youden indices were calculated in order to determinate discriminatory strength. These values vary between 0.89 and 1.

### GEO datasets of independent studies

Marker panel validation was performed on microarray datasets downloaded from Gene Expression Omnibus database. The microarray dataset GSE8671 [20] by *Sabates-Bellver et* *al*. was used which compared the transcriptomes of 32 prospectively collected adenomas with those of normal mucosa from the same individuals. The set of 11 transcripts determined in our microarray study could classify the 32–32 independent adenoma and corresponding normal biopsy samples by 100% specificity and sensitivity. The PCA also showed complete separation between the two sample groups (Figure 1D).

By the same classifiers, 94 CRC and 17 healthy tissue samples from the GSE18105 study [21] could be discriminated both in hierarchical cluster analysis and PCA, with only 1 misclustered normal sample (Figure 1E).

### Array real-time PCR

The array RT-PCR measurements for selected transcript panels were performed on independent biopsy specimens. According to the lowest standard deviation of ΔCT values, 18S ribosomal RNA was chosen as a reference among the seven housekeeping genes placed on the array real-time PCR plate.

PCA figure shows that normal, adenoma and CRC biopsy samples are classified into three distinct groups (Figure 1C).

Discriminant analysis of 11 markers on independent RT-PCR samples showed correct classification for 95.6% of the original grouped cases, and 94.1% of the cross-validated cases (Table 4).

When only 2 sample groups were compared, discriminatory power of the gene panel is also proved to be considerably high during the ROC curve analysis of CRC and normal samples (sensitivity: 100%, specificity: 100%). The adenoma and healthy samples could be clearly separated by 95.8% sensitivity and 95.0% specificity values. In case of adenoma vs. CRC comparison, the ROC curve analysis showed separation with 95.8% sensitivity and specificity.

### Discrimination between high-grade dysplastic adenoma and early CRC samples

The set of 11 classifiers could classify the 24 high-grade dysplastic adenoma and the 24 early CRC (stage Dukes A or B) samples analyzed on microarrays by 83.3% specificity and 100% sensitivity (Figure 3A). This marker set was also suitable for discrimination between high-grade dysplastic adenoma (n = 11) and early cancer (n = 10) samples in real-time PCR analysis.

The hierarchical cluster diagram of the real-time PCR samples represents that all the 10 CRC samples were correctly classified, and 3 of the 11 adenoma samples were misclustered (Figure 3C). These samples were adenoma 6, adenoma 10 and adenoma 11 biopsy samples. However samples 6 and 11 were found to be misclassified as during a patient follow up they were rediagnosed as *in situ* carcinoma (Figure 3D, E). Application of ROC statistic showed even higher differentiation since 100% sensitivity and 90.9% specificity observed in the comparison of samples. Red highlight refers to 6 and 11 adenoma samples which were above or near to the threshold. Green highlight refers to adenoma 10 samples which were clustered with CRC samples but ROC statistic shows clear separation from that group (Figure 3B, D). After patient follow the aforementioned samples transferred into CRC group and new multiple logistic regression was applied. Comparison of 9 high-grade dyslpalstic adenoma 12 and early cancer resulted 100% sensitivity and 100% specificity (Figure 3D), thereby optimize sensitivity (100%) and specificity (90.9%) of original sample classification (Figure 3B).

## Discussion

In this study a characteristic transcript set was determined which is specific for the colorectal dysplasia-carcinoma transition using whole genomic microarray in 53 biopsy samples. In order to test the differentiation power of the discriminatory gene panel, an additional 94 microarrays with independent colonic biopsy specimen and microarray datasets downloaded from the Gene Expression Omnibus were also analyzed. With further validation conducted by array real-time PCR cards that contained the characteristic transcript panel. The identified set of 11 transcripts can be used for separation of CRC, adenoma and normal biopsy samples, moreover it is suitable for discrimination between high-grade dysplastic adenoma and early stage CRC cases by high specificity and sensitivity.

The use of whole genomic microarray analyses represents an important tool for high-throughput gene expression screening, but equipment and reagent costs do not qualify it as for a cost effective diagnostic tool. Therefore quantitative array real-time PCR cards with assays for selected set of classifiers offer a more viable alternative for diagnostic application with lower costs and automation possibility for the whole process from RNA isolation to the RT-PCR analysis [22].

The current method of determining colorectal cancers and adenomas is histological analysis. Colon biopsy specimens are evaluated from 4–5 pieces of small sections of 3–5 µm thick taken from different areas of the colon. However critical areas may remain hidden in the uncut specimen block or due to inadequate orientation including aberrant crypt foci in hyperplastic polyps, in situ carcinoma in adenomas, dysplastic areas and carcinomas in long-time IBD specimens [23]–[24]. In this study, whole biopsy specimens containing mixed cell populations were applied for mRNA expression microarray and real-time PCR analysis in order to overcome the potential sampling errors of conventional histological analysis. Though histological laser microdissection can provide accurate cell type specific information, its major limitation is the need of a very skilled operator, which does not support it to be a candidate diagnostic tool [25].

Further to this, pathologists recently have to face growing workload due to the increasing demand on cancer screening biopsies, molecular testing for target therapy and the concomitant sub-specialization. Therefore, an alternative but still reliable method for identifying diseased or negative specimens could be of great importance. The automated evaluation of colon biopsy specimens by mRNA expression profiling could be a valid approach since much of the methodology, preparation and the analysis procedure are already available.

Furthermore, the mRNA expression analysis gives us an insight into altered cellular functions beyond the microscopic level. This information might be related to the biological behaviour of tumors and/or the expression of therapeutic targets, e.g. growth factor receptors. Also the expression of metastasis related genes and those involved in tumor invasiveness may be identified.

The set of 11 classifiers determined in our study showed considerably high discriminatory power on the microarray datafiles of previous studies in CRC vs. normal and in adenoma vs. normal comparisons. *In silico* results suggest that the identified transcript panel can be used as general discriminative markers for colorectal cancer and polyps. Only datasets with CRC and normal, respectively adenoma and normal biopsy samples can be downloaded from Gene Expression Omnibus database which applied Affymetrix HGU133 Plus 2.0. microarray system. To our knowledge, this study is the first whole genomic oligonucleotide microarray study containing CRC, adenoma and normal biopsy samples together available in GEO which can be suitable for the identification of discriminatory transcripts even between early stage CRC and high-grade dysplastic adenoma tissues. The common pre-processing of the data files from different studies resulted in a clear separation of not only diseased and normal samples, but of adenoma and CRC samples as well. However, the datasets of the different studies are difficult to handle together as the differences of sample preparation can distort the results: this case can cause the overestimation of the efficacy of adenoma and CRC discrimination.

Among the 11 discriminatory transcripts, except COL12A1, ten (namely IL8, MMP3, IL1B, CHI3L1, GREM1, IL1RN, CXCL1, CXCL2, CA7 and SLC7A5) are thought to be associated with colorectal carcinogenesis and progression. In accordance with our findings, 7 of them, such as IL8, CHI3L1, CXCL1, CXCL2, MMP3, SLC7A5 and CA7, were found to be differentially expressed in CRC compared to normal tissue in previous microarray studies [5]–[6], [9]–[10], [12], [26]–[31]. CA7 [29] was also found to be downregulated not only in carcinoma, but in adenoma samples.

Interleukin 8 (IL8) promotes cell proliferation and migration of human colon carcinoma cells through metalloproteinase-cleavage proHB-EGF [32]. The expression of SLC7A5 cationic amino acid transporter was also found to be significantly associated with cell proliferation and angiogenesis [33], moreover it seems to play an important role in enhancing the tumor growth in vivo [34]. The secreted interleukin-like Gro-alpha oncogene (CXCL1) and matrix-metalloproteinase 3 (MMP3) promote tumor initiation and growth (21–22), while chitinase 3 like-1 (CHI3L1) can protect cancer or/and stromal cells against apoptosis [35]. Elevated expression of interleukin 1 beta (IL1B) mRNA increases the risk of non-small cell lung cancer [36]. Although, it is known that IL1B polymorphisms are associated with tumor recurrence in stage II colon cancers [37], the function of this gene has not been clarified in CRC. Gremlin 1 (GREM1) as an antagonist of bone morphogenic proteins, has been shown to regulate early development and tumorigenesis. It was overexpressed in various human tumors and plays an oncogenic role especially in carcinomas including CRC [38]. In previous studies, a highly significant upregulation of CXCL2 chemokine was found in CRC compared to normal colonic mucosa which could be already detected also in benign adenoma referring to the involvement of CXCL2 in the dysplasia-carcinoma transition [39].

In summary, this study identified a set of 11 discriminatory transcripts which could correctly classify not just normal, adenoma and CRC biopsies, but high-grade dysplastic adenoma and early stage CRC samples, even if using a large independent sample set. Although 10 of the 11 discriminatory genes are already known to be associated with CRC, these markers as a combined discriminative set are firstly applied in this study. The identified set of 11 markers was proved to be a highly specific and sensitive discriminator of the colorectal dysplasia-carcinoma transition which is of great clinical importance regarding the early diagnosis of CRC. These markers can establish the basis of gene expression based diagnostic classification of benign and malignant colorectal diseases and of development of diagnostic real-time PCR cards, furthermore they are to be utilized for prospective biopsy screening both at mRNA and protein levels.

## Supporting Information

Table S1 **Supplementary table of the collected and analyzed samples.**
(DOC)
