# Supplementary material for: Dysplasia-Carcinoma Transition Specific Transcripts in Colonic Biopsy Samples
Source: PLoS One. 2012 Nov 14;7(11):e48547. doi: 10.1371/journal.pone.0048547 (PMC3498283; doi:10.1371/journal.pone.0048547)
Supplement: Table S1 — Supplementary table of the collected and analyzed samples. (DOC) [file pone.0048547.s001.doc]

| **Sample ID** | **Sample**  **type** | **Sex** | **Age** | **Localization** | **Histology** | **TNM** | **Grade** | **Dukes** | **Dysplasia** | **Affymetrix** | **Array RT-PCR** |
| --- | --- | --- | --- | --- | --- | --- | --- | --- | --- | --- | --- |
| **Normal** | | | | | | | | | | | |
| 2622 | biopsy | F | 30 | sigma | normal |  |  |  |  | N2622.CEL |  |
| 2642 | biopsy | M | 57 | sigma | normal |  |  |  |  | N2642.CEL |  |
| 2647 | biopsy | M | 36 | coecum | normal |  |  |  |  | N2647.CEL | + |
| 2664 | biopsy | F | 49 | sigma | normal |  |  |  |  | N2664.CEL | + |
| 2668 | biopsy | F | 65 | rectum | normal |  |  |  |  | N2668.CEL | + |
| 2675 | biopsy | F | 46 | sigma | normal |  |  |  |  | N2675.CEL |  |
| 2689 | biopsy | M | 54 | sigma | normal |  |  |  |  | N2689.CEL |  |
| 2691 | biopsy | F | 77 | sigma | normal |  |  |  |  | N2691.CEL | + |
| 2701 | biopsy | M | 27 | - | normal |  |  |  |  | N2701.CEL |  |
| 2770 | biopsy | F | 82 | sigma | normal |  |  |  |  | N2770.CEL | + |
| 2771 | biopsy | F | 62 | sigma | normal |  |  |  |  | N2771.CEL | + |
| 2781 | biopsy | M | 74 | sigma | normal |  |  |  |  | N2781.CEL | + |
| 2785 | biopsy | M | 71 | sigma | normal |  |  |  |  | N2785.CEL |  |
| 2791 | biopsy | M | 77 | sigma | normal |  |  |  |  | - | + |
| 2801 | biopsy | F | 68 | sigma | normal |  |  |  |  | - | + |
| 2813 | biopsy | M | 54 | sigma | normal |  |  |  |  | - | + |
| 2851 | biopsy | M | 63 | sigma | normal |  |  |  |  | - | + |
| 3008 | biopsy | F | 54 | sigma, rectum | normal |  |  |  |  | N3008.CEL | + |
| 3020 | biopsy | F | 62 | colcn asc, sigma | normal |  |  |  |  | N3020.CEL |  |
| 3035 | biopsy | M | 22 | colon asc, desc, sigma | normal |  |  |  |  | N3035.CEL |  |
| 3038 | biopsy | F | 73 | colon asc, sigma | normal |  |  |  |  | N3038.CEL | + |
| 3039 | biopsy | F | 75 | sigma | normal |  |  |  |  | N3039.CEL |  |
| 3040 | biopsy | M | 41 | - | normal |  |  |  |  | N3040.CEL |  |
| 3048 | biopsy | F | 43 | sigma, rectum | normal |  |  |  |  | N3048.CEL |  |
| 3054 | biopsy | M | 41 | sigma, rectum | normal |  |  |  |  | N3054.CEL |  |
| 3057 | biopsy | M | 30 | colon asc, rectum | normal |  |  |  |  | N3057.CEL | + |
| 3062 | biopsy | M | 66 | sigma | normal |  |  |  |  | N3062.CEL |  |
| 3065 | biopsy | F | 62 | sigma | normal |  |  |  |  | N3065.CEL | + |
| 3069 | biopsy | F | 61 | sigma | normal |  |  |  |  | N3069.CEL |  |
| 3090 | biopsy | F | 40 | - | normal |  |  |  |  | N3090.CEL | + |
| 3103 | biopsy | M | 41 | sigma, rectum | normal |  |  |  |  | N3103.CEL | + |
| 3106 | biopsy | F | 47 | - | normal |  |  |  |  | N3106.CEL | + |
| 3108 | biopsy | M | 67 | - | normal |  |  |  |  | N3108.CEL | + |
| 3109 | biopsy | F | 40 | colon desc, rectum | normal |  |  |  |  | N3109.CEL |  |
| 3112 | biopsy | F | 44 | colon asc, transv, desc, sigma | normal |  |  |  |  | N3112.CEL | + |
| 3114 | biopsy | F | 31 | colon asc, sigma | normal |  |  |  |  | N3114.CEL |  |
| 3115 | biopsy | F | 82 | sigma, rectum | normal |  |  |  |  | N3115.CEL |  |
| 3118 | biopsy | F | 33 | - | normal |  |  |  |  | N3118.CEL |  |
| 3126 | biopsy | F | 64 | sigma, rectum | normal |  |  |  |  | N3126.CEL |  |
| 3129 | biopsy | F | 64 | coecum, sigma | normal |  |  |  |  | N3129.CEL |  |
| 3140 | biopsy | F | 45 | sigma | normal |  |  |  |  | N3140.CEL |  |
| 3142 | biopsy | F | 51 | sigma | normal |  |  |  |  | N3142.CEL |  |
| **Low-grade dysplastic adenoma** | | | | | | | | | | | |
| 2619 | biopsy | F | 51 | sigma | tubulovillous adenoma |  |  |  | low-grade | AL2619.CEL | + |
| 2623 | biopsy | F | 61 | coecum | tubulovillous adenoma |  |  |  | low-grade | AL2623.CEL | + |
| 2678 | biopsy | M | 71 | colon desc | tubular adenoma |  |  |  | low-grade | AL2678.CEL | + |
| 2796 | biopsy | F | 50 | sigma | villous adenoma |  |  |  | low-grade | AL2796.CEL | + |
| 2830 | biopsy | F | 62 | rectum | tubular adenoma |  |  |  | low-grade | - | + |
| 2863 | biopsy | F | 88 | colon transv | tubulovillous adenoma |  |  |  | low-grade | AL2863.CEL | + |
| 2870 | biopsy | F | 78 | hepatic flexure | tubular adenoma |  |  |  | low | - | + |
| 2902 | biopsy | F | 62 | rectum | tubular adenoma |  |  |  | low | - | + |
| 2937 | biopsy | M | 57 | rectum | tubular adenoma |  |  |  | low-grade | AL2937.CEL | + |
| 2957 | biopsy | M | 59 | transversum | tubular adenoma |  |  |  | low-grade | AL2957.CEL | + |
| 2964 | biopsy | M | 77 | sigma | tubular adenoma |  |  |  | low-grade | AL2964.CEL | + |
| 2982 | biopsy | M | 58 | sigma | tubular adenoma |  |  |  | low-grade | AL2982.CEL | + |
| 2989 | biopsy | F | 73 | colon asc | tubular adenoma |  |  |  | low-grade | AL2989.CEL | + |
| 3001 | biopsy | F | 75 | sigma | tubulovillous adenoma |  |  |  | low-grade | AL3001.CEL |  |
| 3026 | biopsy | M | 83 | colon asc | tubular adenoma |  |  |  | low-grade | AL3026.CEL |  |
| 3045 | biopsy | F | 65 | sigma | tubulovillous adenoma |  |  |  | low-grade | AL3045.CEL |  |
| 3049 | biopsy | M | 54 | rectosigmoideal | tubular adenoma |  |  |  | low-grade | AL3049.CEL |  |
| 3111 | biopsy | M | 70 | rectum | tubulovillous adenoma |  |  |  | low-grade | AL3111.CEL |  |
| 3120 | biopsy | M | 66 | rectum | tubulovillous adenoma |  |  |  | low-grade | AL3120.CEL |  |
| **High-grade dysplastic adenoma** | | | | | | | | | | | |
| 2573 | biopsy | F | 59 | rectum | tubulovillous adenoma |  |  |  | high-grade | AH2573.CEL | + |
| 2729 | biopsy | F | 58 | Rectum-sigma | tubulovillous adenoma |  |  |  | high-grade | - | + |
| 2739 | biopsy | M | 62 | rectum | villous adenoma |  |  |  | high-grade | AH2739.CEL | + |
| 2803 | biopsy | M | 43 | sigma | tubular adenoma |  |  |  | high-grade | AH2803.CEL | + |
| 2810 | biopsy | M | 61 | rectum | tubulovillous adenoma |  |  |  | high-grade | AH2810.CEL | + |
| 2821 | biopsy | F | 79 | sigma | tubular adenoma |  |  |  | high-grade | AH2821.CEL |  |
| 2916 | biopsy | M | 67 | rectum | villous adenoma |  |  |  | high-grade | AH2916.CEL | + |
| 2945 | biopsy | F | 87 | rectum | tubular adenoma |  |  |  | high-grade | AH2945.CEL |  |
| 2952 | biopsy | M | 62 | colon asc | tubular adenoma |  |  |  | high-grade | AH2952.CEL | + |
| 3051 | biopsy | F | 75 | sigma | tubular adenoma |  |  |  | high-grade | AH3051.CEL |  |
| 3059 | biopsy | M | 69 | rectum | tubulovillous adenoma |  |  |  | high-grade | AH3059.CEL | + |
| 3139 | biopsy | M | 69 | coecum | tubulovillous adenoma |  |  |  | high-grade | AH3139.CEL | + |
| 3147 | biopsy | F | 49 | rectum | tubulovillous adenoma |  |  |  | high-grade | AH3147.CEL | + |
| 3151 | biopsy | F | 78 | colon transv | villous adenoma |  |  |  | high-grade | AH3151.CEL | + |
| **CRC Dukes A, B** | | | | | | | | | | | |
| 2681 | biopsy | M | 67 | rectum | adenocarcinoma | T1N0M0 | G2 | A |  | B2681.CEL | + |
| 2682 | biopsy | F | 80 | colon asc | adenocarcinoma | T3N0M0 | G2 | B2 |  | B2682.CEL | + |
| 2707 | biopsy | F | 63 | rectum | adenocarcinoma | T3N0M0 | G2 | B2 |  | B2707.CEL | + |
| 2724 | biopsy | F | 76 | coecum | adenocarcinoma | T3N0M0 | G2 | B2 |  | B2724.CEL | + |
| 2849 | biopsy | F | 80 | colon asc | adenocarcinoma | T3N0M0 | G2 | B2 |  | B2849.CEL | + |
| 2865 | biopsy | M | 85 | coecum | adenocarcinoma | T2N0M0 | G2 | B1 |  | B2865.CEL | + |
| 2900 | biopsy | M | 79 | coecum | adenocarcinoma | T3N0M0 | G2 | B2 |  | B2900.CEL | + |
| 2925 | biopsy | F | 76 | colon transv. | adenocarcinoma | T3N0M0 | G2 | B2 |  | B2925.CEL |  |
| 2927 | biopsy | F | 65 | rectum | adenocarcinoma | T2N0M0 | G1 | B1 |  | B2927.CEL |  |
| 2940 | biopsy | F | 65 | rectum | adenocarcinoma | T3N0M0 | G1 | B2 |  | B2940.CEL | + |
| 2956 | biopsy | F | 67 | sigma | adenocarcinoma | T3N0M0 | G2 | B2 |  | B2956.CEL | + |
| 2986 | biopsy | F | 67 | colon asc. | adenocarcinoma | T2N0M0 | G1 | B1 |  | B2986.CEL | + |
| 2991 | biopsy | F | 81 | colon asc. | adenocarcinoma | T3N0M0 | G2 | B2 |  | B2991.CEL |  |
| 3068 | biopsy | M | 73 | flex.lien. | adenocarcinoma | T3N0M0 | G1 | B2 |  | B3068.CEL |  |
| **CRC Dukes C, D** | | | | | | | | | | | |
| 1316 | biopsy | M | 85 | flex. lien. | adenocarcinoma | T3N1M1 | G2 | D |  | 1316 U133 Plus 2.0 .CEL | + |
| 1494 | biopsy | M | 73 | rectum | adenocarcinoma | T3N1M1 | G1 | D |  | 1494 U133 Plus 2.0 .CEL | + |
| 2651 | biopsy | F | 55 | rectum-sigma | adenocarcinoma | T3N2M0 | G2 | C3 |  | D2651.CEL |  |
| 2660 | biopsy | F | 73 | rectum | adenocarcinoma | T3N2M1 | G2 | D |  | D2660.CEL | + |
| 2662 | biopsy | M | 78 | colon asc. | adenocarcinoma | T3N2M1 | G2 | D |  | D2662.CEL |  |
| 2684 | biopsy | F | 74 | coecum | adenocarcinoma | T3N1M0 | G2 | C |  | D2684.CEL |  |
| 2730 | biopsy | M | 65 | rectum | adenocarcinoma | T2N1M0 | G2 | C1 |  | D2730.CEL | + |
| 2751 | biopsy | F | 85 | coecum | adenocarcinoma | T3N1M0 | G2 | C |  | D2751.CEL | + |
| 2765 | biopsy | M | 48 | rectum | adenocarcinoma | T4N2M0 | G2 | C3 |  | D2765.CEL | + |
| 2842 | biopsy | F | 66 | rectum | adenocarcinoma | T3N1M0 | G2 | C |  | D2842.CEL | + |
| 2872 | biopsy | F | 61 | rectum | adenocarcinoma | T3N1M0 | G2 | C2 |  | D2872.CEL | + |
| 2876 | biopsy | M | 70 | rectum | adenocarcinoma | T3N1M0 | G3 | C2 |  | D2876.CEL | + |
| 2884 | biopsy | F | 75 | rectum | adenocarcinoma | T3N1M1 | G2 | D |  | D2884.CEL | + |
| 2903 | biopsy | M | 60 | rectum | adenocarcinoma | T3N1M1 | G2 | D |  | D2903.CEL |  |
| 2936 | biopsy | M | 40 | rectum | adenocarcinoma | T2N3M1 | G2 | D |  | D2936.CEL |  |
| **CRC with unknown stage** | | | | | | | | | | | |
| 2635 | biopsy | F | 62 | colon desc | adenocarcinoma | - | - | - |  | - | + |
| 2720 | biopsy | F | 60 | rectum | adenocarcinoma | - | - | - |  | - | + |
| 2886 | biopsy | M | 78 | sigma | adenocarcinoma | - | - | - |  | - | + |
| 2930 | biopsy | F | 61 | rectum | adenocarcinoma | - | - | - |  | - | + |
